# Supplementary material for: BOA/DHB/Na: An Efficient UV-MALDI Matrix for High-Sensitivity and Auto-Tagging Glycomics
Source: Int J Mol Sci. 2022 Oct 19;23(20):12510. doi: 10.3390/ijms232012510 (PMC9604046; doi:10.3390/ijms232012510)
Supplement: Supplementary file 1 [file ijms-23-12510-s001.zip › ijms-1958397-supplementary.pdf]

Supplementary Materials

BOA/DHB/Na: an Efficient UV-MALDI Matrix for High-Sensitivity and Auto-Tagging Glycomics

Erina Barada, Hiroshi Hinou*

Graduate School of Life Sciece and Faculty of Advanced Life Science, Frontier Research Center for Advanced Material and Life Science, Hokkaido University, N21W11, Sapporo 001-0021, Japan.
*e-mail: hinou@sci.hokudai.ac.jp

List of contents

Figure S1. Chemical structure of sodium adduct ion of SGP-10 and its oxime and imine form.
Figure S2. Morphological differences in each matrix system (analyte is SGP-10)
Figure S3. MALDI-TOFMS spectra of SGP-10 in (a) BOA/DHB/Na matrix and (b) BOA HCl/DHB/Na matrix after overnight incubation at room temperature.
Figure S4. MALDI-TOFMS spectra of SGP-10 in (a) BOADHB/Na matrix and (b) aniline/DHB/Na matrix after overnight incubation at room temperature.
Figure S5. Enlarged view of the main peak area in Figure 4b.
Figure S6. Supporting data of Figure 5.
Figure S7. MALDI-TOF/TOF MS fragmentation patterns of SGP-10 with four different matrix system.
Figure S8. Bar chart showing the amount of the top ten *O*-linked glycans in MPS (among five lots) analyzed with BOA/DHB/Na matrix.
Figure S9. Morphological differences of each matrix system used for profiling of *O*-glycan from MPS.
Figure S10. MALDI-TOFMS spectra from MPS by the workflow in Scheme 1 (with BOA/DHB/Na matrix).
Figure S11. MALDI-TOFMS spectra of the Cotton HILIC eluate from MPS by the workflow in Scheme 1 with DHB/Na matrix.
Figure S12. MALDI-TOFMS spectra of the Cotton HILIC eluate from MPS by the workflow in Scheme 1 with DHB (0.1% TFA) matrix.
Table S1. List of the composition of *O*-glycans assigned by GlycoMod for twenty peaks (*m*/*z*) detected from MPS by the workflow in Scheme 1.
Table S2. Amount list of the twenty *O*-glycans detected from MPS by the workflow in Scheme 1.

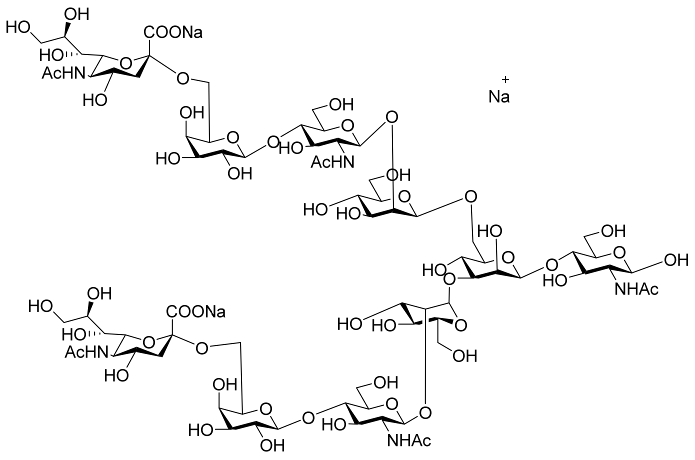

SGP-10 [(M-2H+3Na)+ = 2087]

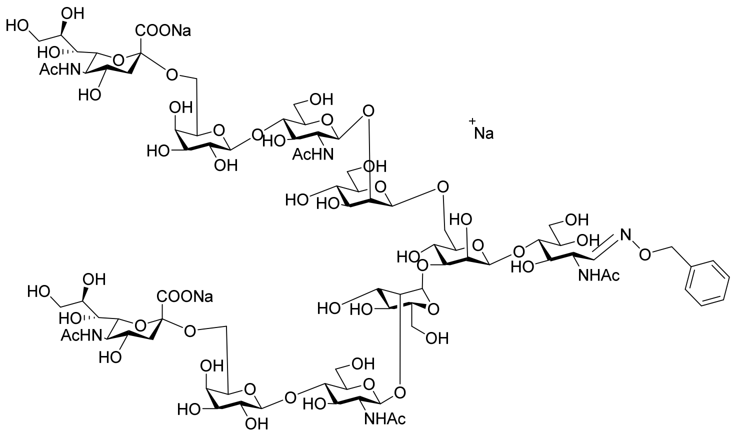

SGP-10-BOA [(M-2H+BOA+3Na)+ = 2192]

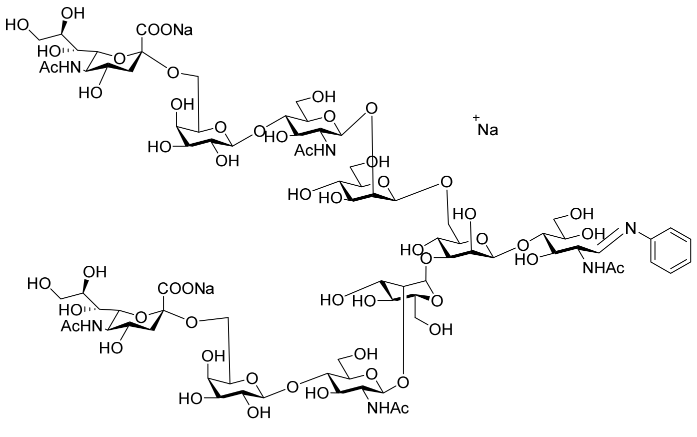

SGP-10-Aniline [(M-2H+Aniline+3Na)+ = 2162]

Figure S1. Chemical structure of sodium adduct ion of SGP-10 and its oxime and imine form.

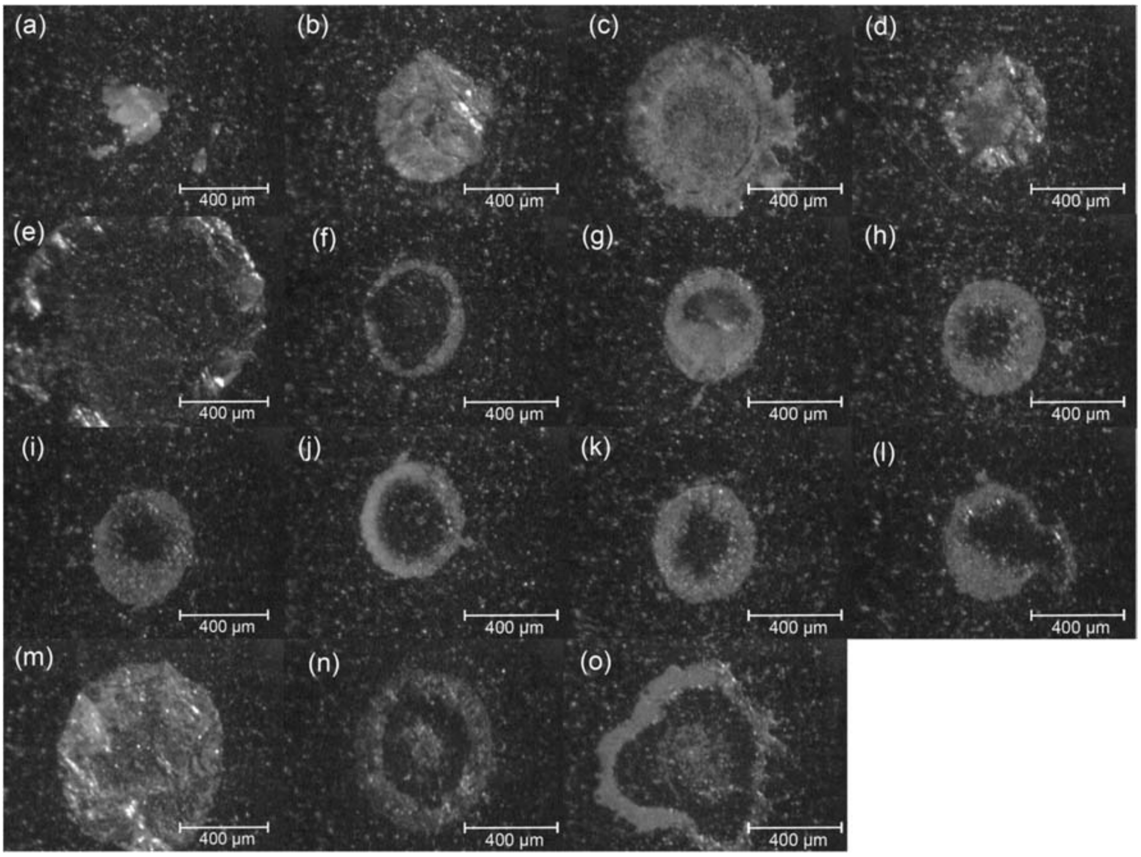

Figure S2. Morphological differences in each matrix system (analyte is SGP-10); (a) BOA/DHB/Na matrix (dried overnight at room temperature), (b) BOA HCl/DHB/Na matrix (dried overnight at room temperature), (c) aniline/DHB/Na matrix (dried overnight at room temperature), (d) NMA/DHB/Na matrix(dried overnight at room temperature), (e) DHB (0.1% TFA) (dried with hairdryer), (f) BOA/DHB/Na matrix (dried 1 hour at room temperature), (g) BOA/DHB/Na matrix (dried 3 hour at room temperature), (h) BOA/DHB/Na matrix (dried 1 hour at 60°C), (i) BOA/DHB/Na matrix (dried 1 hour at 50°C), (j) BOA/DHB/Na matrix (dried 1 hour at 40°C), (k) BOA/DHB/Na matrix (dried 30 minutes at 60°C), (l) BOA/DHB/Na matrix (dried 30 minutes at 50°C), (m) BOA HCl/DHB/Na matrix (dried 1 hour at 60°C), (n) aniline/DHB/Na matrix (dried 1 hour at 60°C), (o) NMA/DHB/Na matrix (dried 1 hour at 60°C).

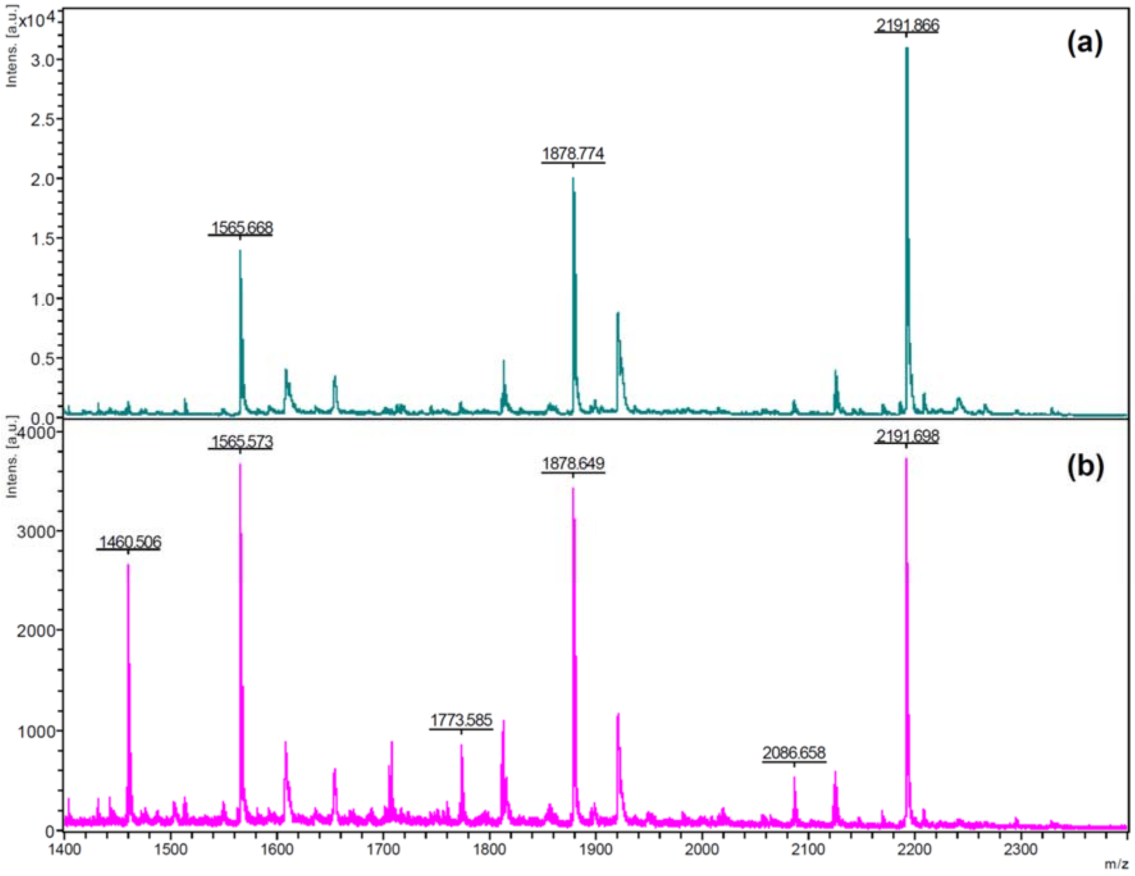

Figure S3. MALDI-TOFMS spectra of SGP-10 in (a) BOA/DHB/Na matrix and (b) BOA HCl/DHB/Na matrix after overnight incubation at room temperature.

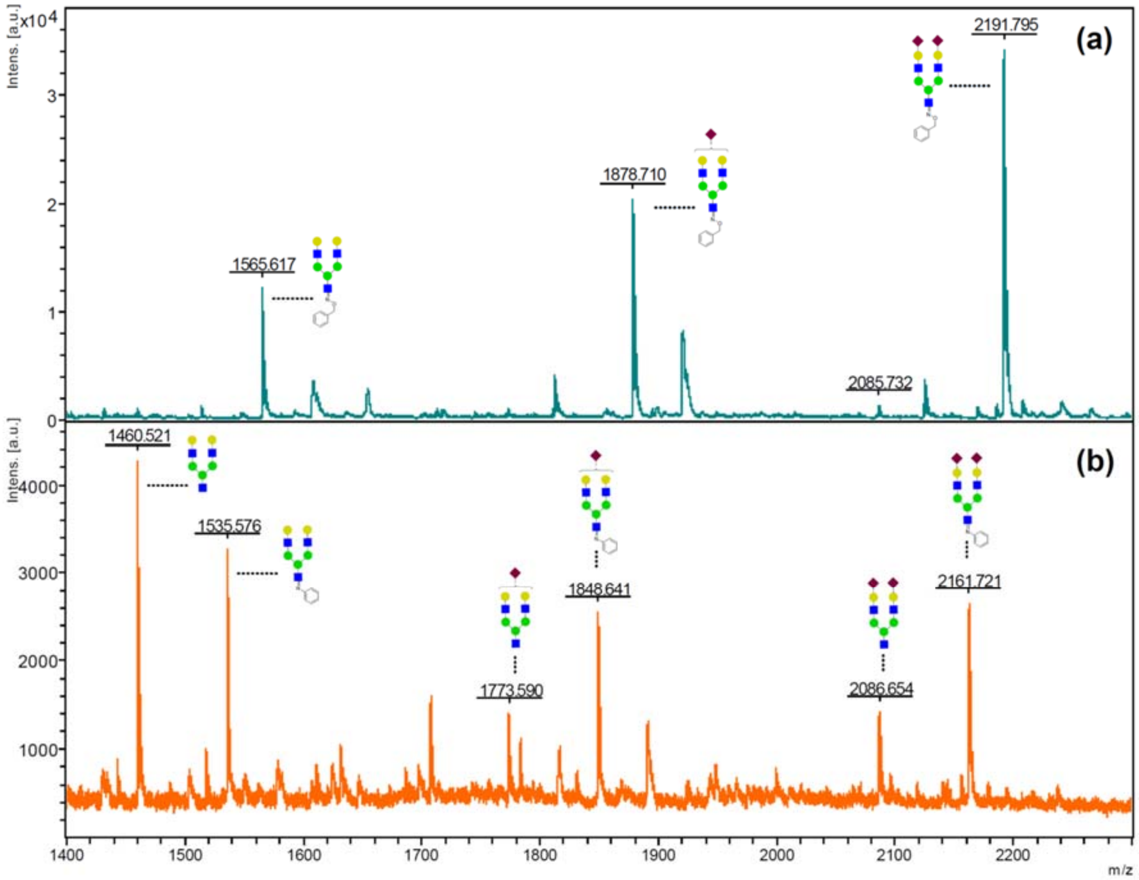

Figure S4. MALDI-TOFMS spectra of SGP-10 in (a) BOA/DHB/Na matrix and (b) aniline/DHB/Na matrix after overnight incubation at room temperature.

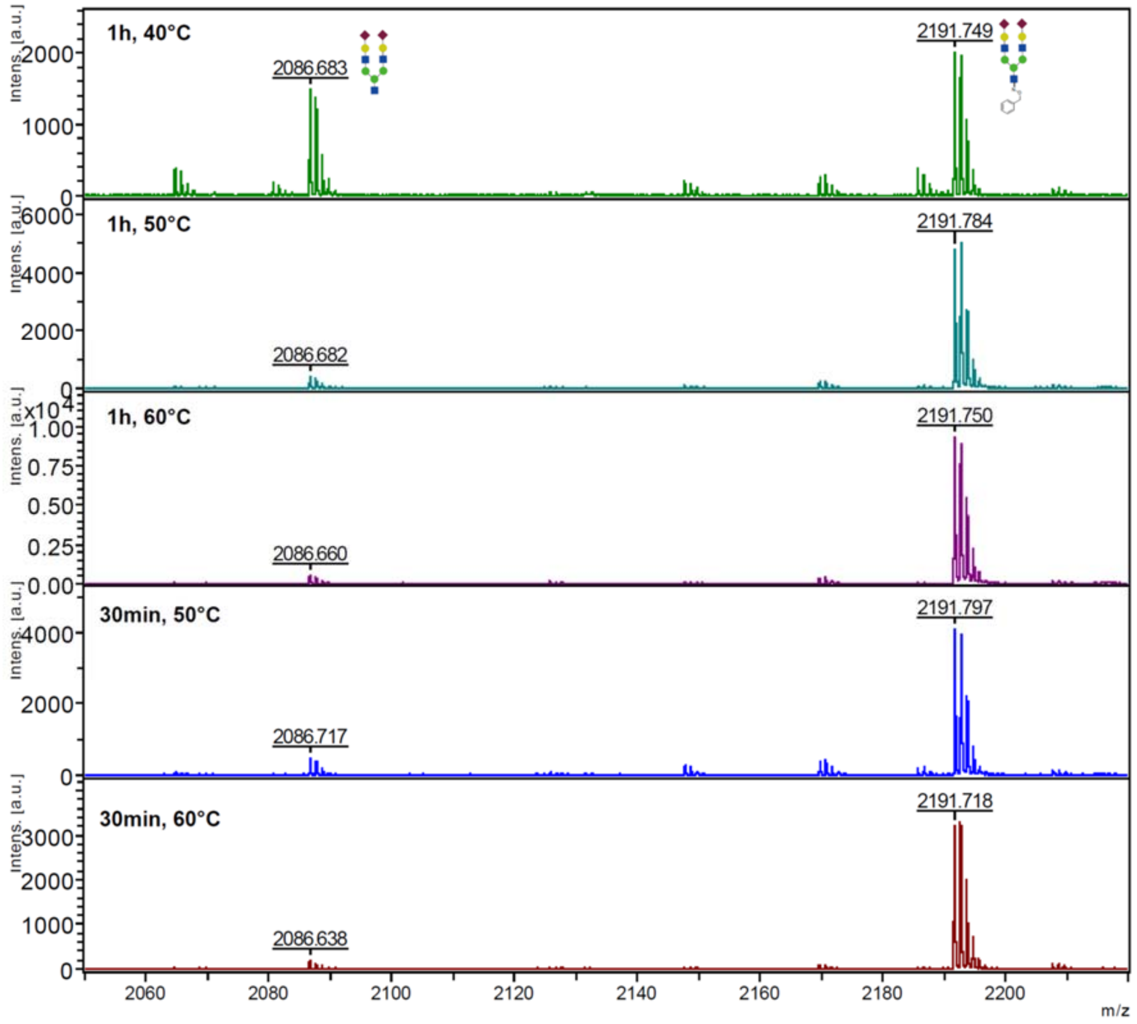

Figure S5. Enlarged view of the main peak area in Figure 4b.

(a)

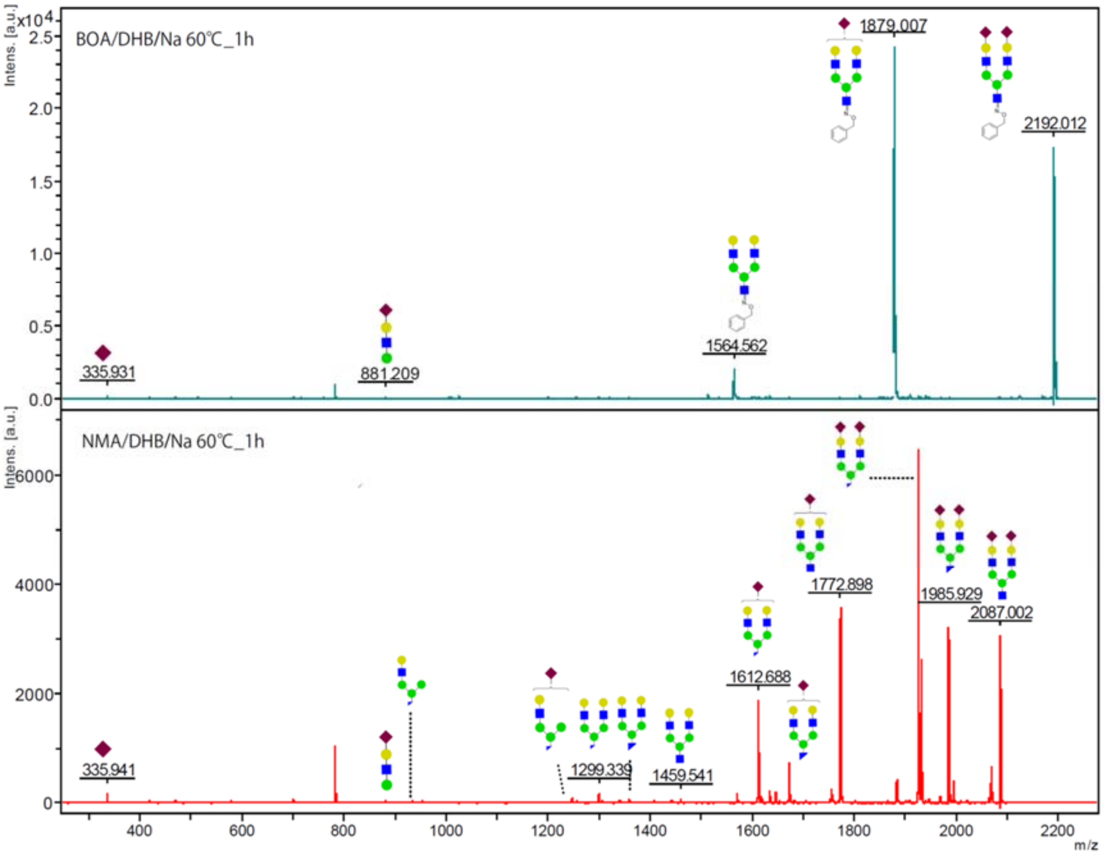

(b)

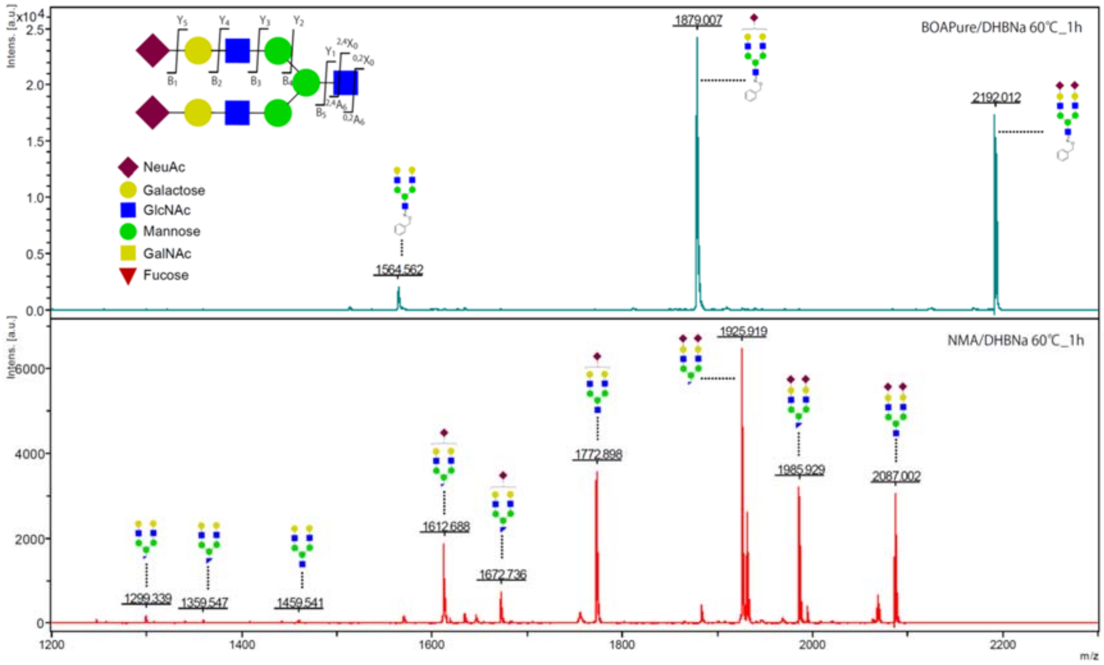

Figure S6. Supporting data of Figure 5; (a) Peak picked spectrum, (b) enlarged view of the main peak area in Figure 5.

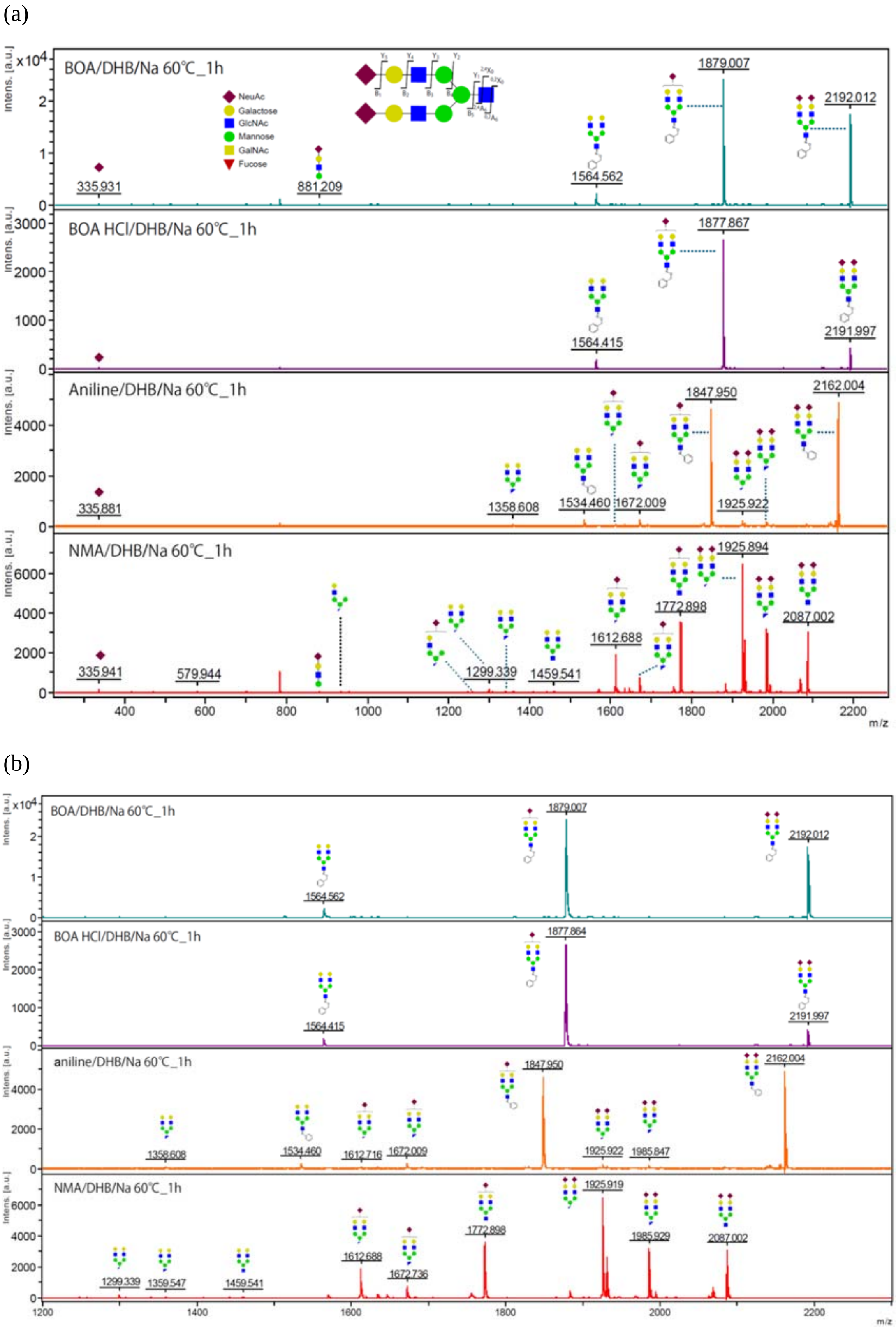

Figure S7. MALDI-TOF/TOF MS fragmentation patterns of SGP-10 with four different matrix system; (a) *m/z* 200-2300, (b) *m/z* 1200-2300.

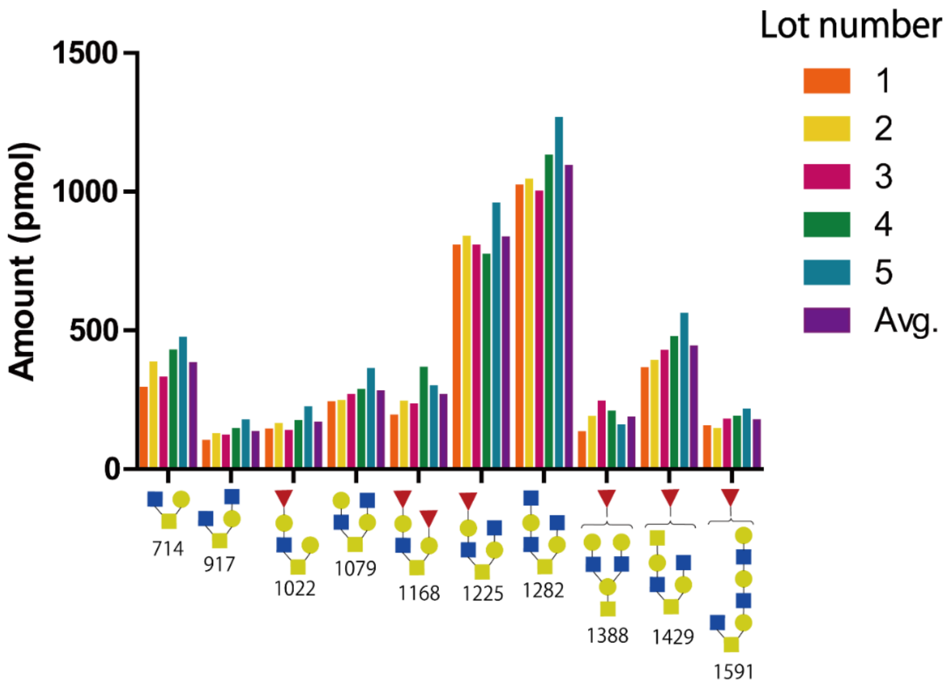

Figure S8. Bar chart showing the amount of the top ten *O*-linked glycans in MPS (among five lots) analyzed with BOA/DHB/Na matrix.

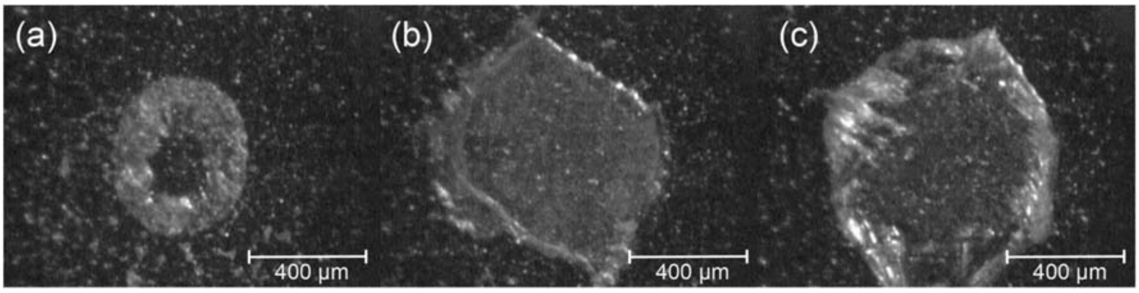

Figure S9. Morphological differences of each matrix system used for profiling of *O*-glycan from MPS; (a) BOA/DHB/Na matrix (dried 1 hour at 60°C), (b) DHB/Na matrix (dried with hairdryer), (c) DHB (0.1% TFA) (dried with hairdryer).

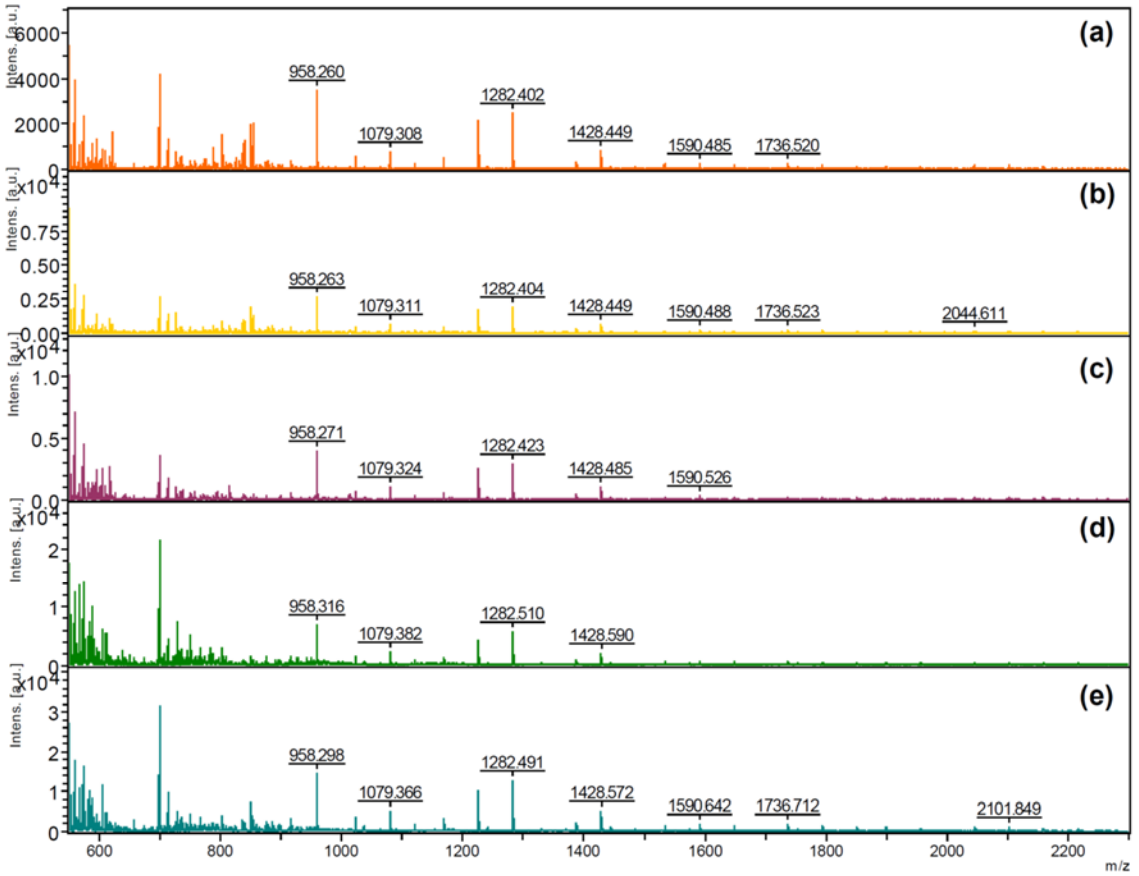

Figure S10. MALDI-TOFMS spectra from MPS by the workflow in Scheme 1 (with BOA/DHB/Na matrix); (a)-(e) Lot number 1-5, respectively.

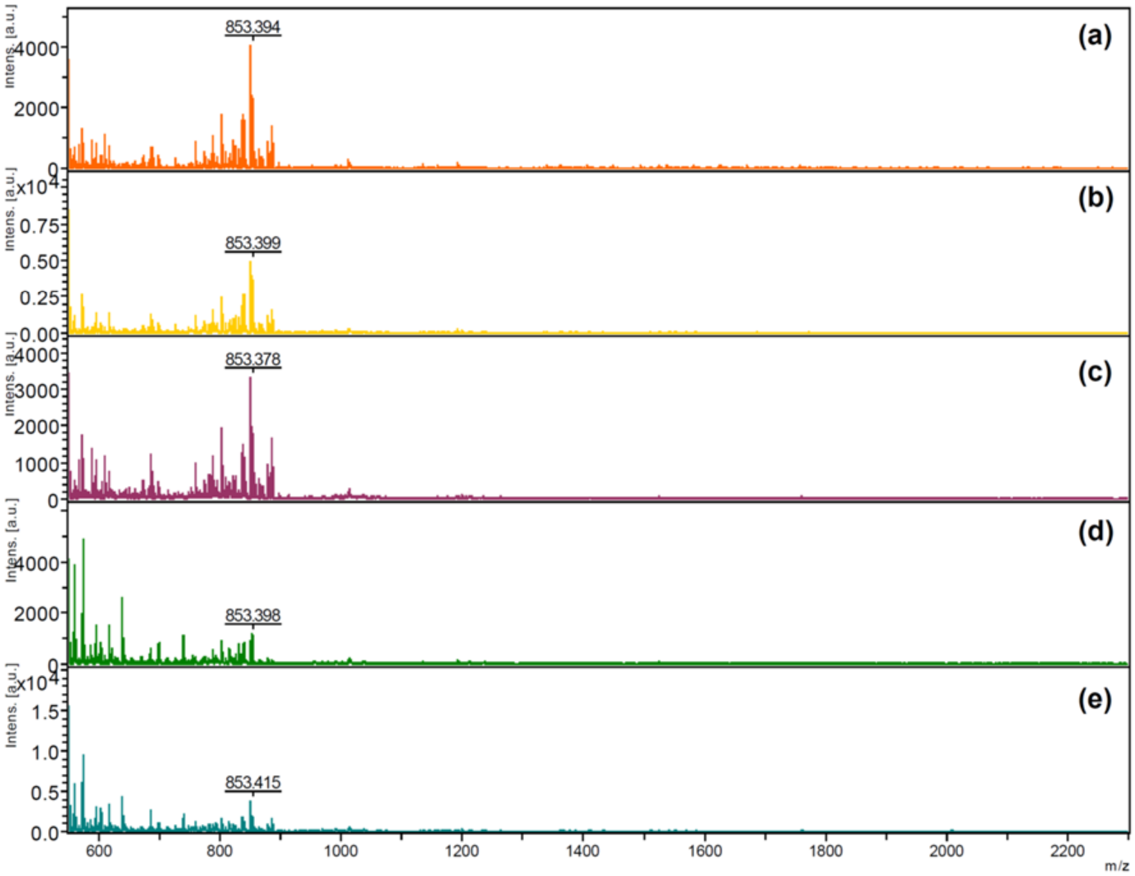

Figure S11. MALDI-TOFMS spectra of the Cotton HILIC eluate from MPS by the workflow in Scheme 1 with DHB Na matrix; (a)-(e) Lot number 1-5, respectively.

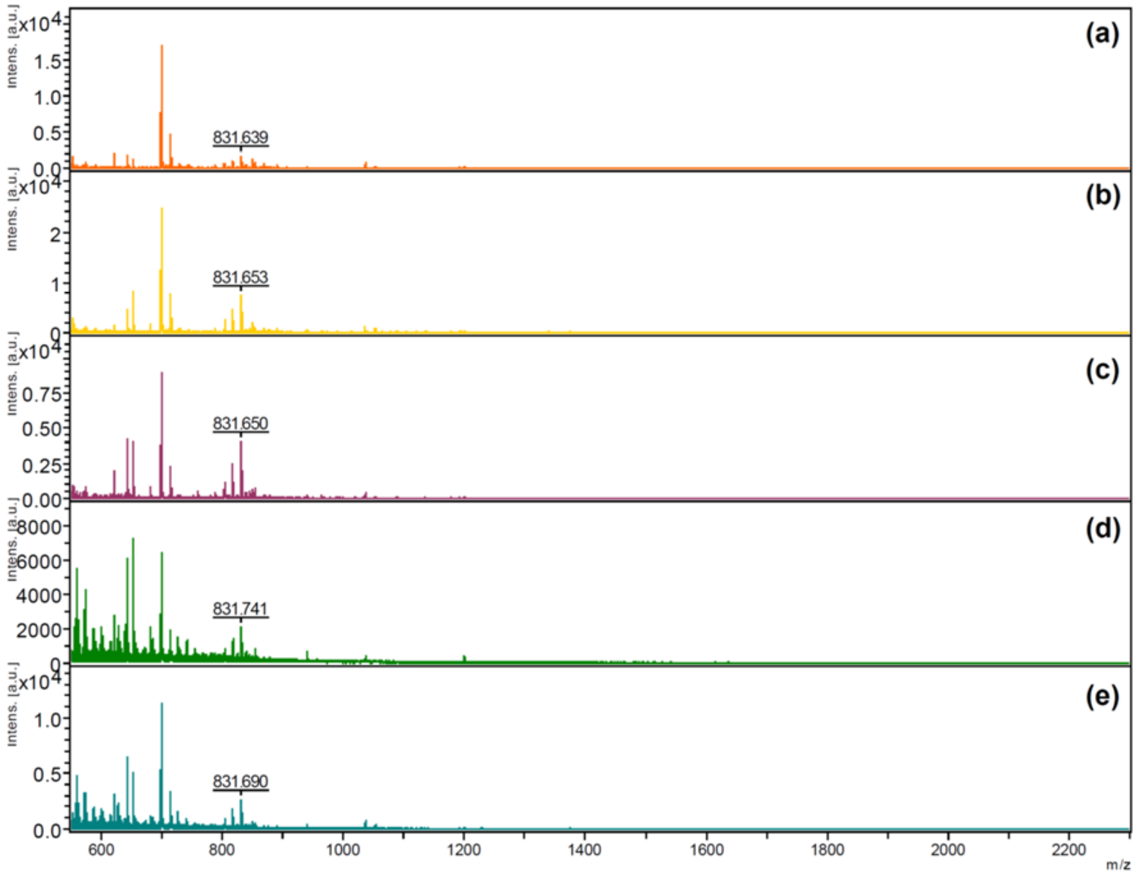

Figure S12. MALDI-TOFMS spectra of the Cotton HILIC eluate from MPS by the workflow in Scheme 1 with DHB TFA matrix; (a)-(e) Lot number 1-5, respectively.

Table S1. List of the composition of *O*-glycans assigned by GlycoMod for twenty peaks (*m*/*z*) detected from MPS by the workflow in Scheme 1.

peak	*m*/*z*	Glycan composition	
1	657.02	$(Hex)_1$ $(HexNAc)_1$ $(Deoxyhexose)_1$	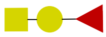
2	714.08	$(Hex)_1$ $(HexNAc)_2$	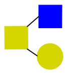
3	860.21	$(Hex)_1$ $(HexNAc)_2$ $(Deoxyhexose)_1$	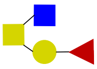
4	876.22	$(Hex)_2$ $(HexNAc)_2$	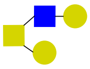
5	917.26	$(Hex)_1$ $(HexNAc)_3$	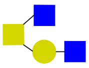
6	1022.32	$(Hex)_2$ $(HexNAc)_2$ $(Deoxyhexose)_1$	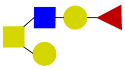
7	1079.36	$(Hex)_2$ $(HexNAc)_3$	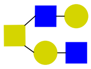
8	1120.39	$(Hex)_1$ $(HexNAc)_4$	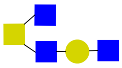
9	1168.41	$(Hex)_2$ $(HexNAc)_2$ $(Deoxyhexose)_2$	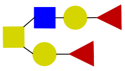
10	1225.46	$(Hex)_2$ $(HexNAc)_3$ (Deoxyhexose)1	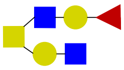
11	1282.49	$(Hex)_2$ $(HexNAc)_4$	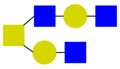
12	1387.54	$(Hex)_3$ $(HexNAc)_3$ $(Deoxyhexose)_1$	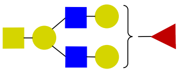
13	1428.57	$(Hex)_2$ $(HexNAc)_4$ $(Deoxyhexose)_1$	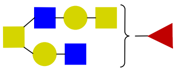
14	1533.61	$(Hex)_3$ $(HexNAc)_3$ $(Deoxyhexose)_2$	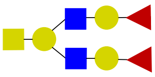
15	1590.64	$(Hex)_3$ $(HexNAc)_4$ $(Deoxyhexose)_1$	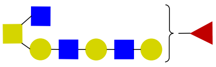
16	1647.67	$(Hex)_3$ $(HexNAc)_5$	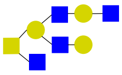
17	1736.71	$(Hex)_3$ $(HexNAc)_4$ $(Deoxyhexose)_2$	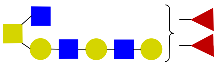
18	1793.73	$(Hex)_3$ $(HexNAc)_5$ $(Deoxyhexose)_1$	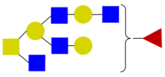
19	2044.83	$(Hex)_4$ $(HexNAc)_4$ $(Deoxyhexose)_3$	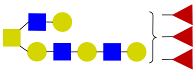
20	2101.85	$(Hex)_4$ $(HexNAc)_5$ $(Deoxyhexose)_2$	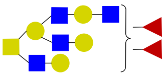

Table S2. Amount list of the twenty *O*-glycans from MPS detected by the workflow processing in Scheme 1.

		pmol					
peak	*m/z*	Lot 1	Lot 2	Lot 3	Lot 4	Lot 5	Average
1	657.02	-	-	-	-	96.23	19.25
2	714.08	293.62	385.10	330.33	427.59	474.70	382.27
3	860.21	-	-	-	-	70.79	14.16
4	876.22	77.67	-	-	-	128.23	41.18
5	917.26	101.31	126.65	121.93	144.27	175.75	133.98
6	1022.32	143.24	163.59	138.41	173.46	223.02	168.34
7	1079.36	241.29	245.97	268.32	285.31	360.75	280.33
8	1120.39	-	-	-	-	136.71	27.34
9	1168.41	193.49	243.31	233.40	365.47	299.97	267.13
10	1225.46	806.10	838.20	806.05	772.65	957.57	836.12
11	1282.49	1021.99	1044.58	1000.62	1130.32	1265.48	1092.60
12	1387.54	132.97	188.16	244.16	208.71	158.12	186.42
13	1428.57	364.05	390.53	427.39	476.72	559.95	443.73
14	1533.61	-	-	-	155.55	165.84	64.28
15	1590.64	154.75	143.96	179.99	189.28	215.96	176.79
16	1647.67	-	123.87	-	168.38	172.65	92.98
17	1736.71	176.50	176.44	-	191.02	237.00	156.19
18	1793.73	-	-	-	175.22	203.67	75.78
19	2044.83	-	142.32	-	141.28	180.18	92.76
20	2101.85	-	118.74	-	176.63	179.09	94.89
